# A metal–organic framework nanocomposite with oxidation and near-infrared light cascade response for bacterial photothermal inactivation

**DOI:** 10.3389/fchem.2022.1044931

**Published:** 2022-10-31

**Authors:** Christopher Dorma Momo, Yuan Zhou, Lanxin Li, Weisheng Zhu, Luyao Wang, Xingping Liu, Wei Bing, Zhijun Zhang

**Affiliations:** ^1^ Key Laboratory of Surface and Interface Science of Polymer Materials of Zhejiang Province, Department of Chemistry, Zhejiang Sci-Tech University, Hangzhou, China; ^2^ Department of Pharmacy, Taihe Hospital, Hubei University of Medicine, Shiyan, Hubei, China; ^3^ College of Pharmacy, Hubei University of Traditional Chinese Medicine, Wuhan, China; ^4^ School of Chemistry and Life Science, Changchun University of Technology, Changchun, China; ^5^ School of Pharmaceutical Science, University of South China, Hengyang, China

**Keywords:** metal–organic framework, cascade response, antibacterial materials, photothermal therapy, nanodrug

## Abstract

Photothermal treatment is an effective and precise bacterial disinfection method that can reduce the occurrence of bacterial drug resistance. However, most conventional photothermal treatment strategies have the problem that the photothermal response range does not match the infection area. Herein, a metal–organic framework (MOF) nanocomposite responding to the oxidation state of the bacterial infection microenvironment was constructed for near-infrared (NIR) photothermal bacterial inactivation. In this strategy, the MOF was used as a nanocarrier to load tetramethylbenzidine (TMB) and horseradish peroxidase (HPR). The high oxidation state of the bacterial infection microenvironment can trigger the enzyme-catalyzed reaction of the nanocomposite, thereby generating oxidation products with the NIR photothermal effect for bacterial disinfection. The synthesis and characterization of the nanocomposite, oxidation state (H_2_O_2_) response effect, photothermal properties, and antibacterial activities were systematically studied. This study provides a new idea for building a precision treatment system for bacterial infection.

## Introduction

Bacterial infection seriously threatens human life and health. As a traditional medicine for treating bacterial infections, antibiotics have saved countless lives. However, the use of antibiotics will lead to the emergence of bacterial resistance, which greatly reduces the therapeutic effect of antibiotics and even makes antibiotics ineffective (Laxminarayan et al., 2013; Mamun et al., 2021). The abuse of antibiotics in recent years has accelerated the emergence of bacterial drug resistance. Unfortunately, the speed at which we develop new antibiotics is far lower than the speed at which bacterial drug resistance develops (Hutchings et al., 2019). According to the World Health Organization (WHO), around 7 00,000 people die of drug-resistant bacterial infections every year worldwide. If effective measures are not taken, it is estimated that 10 million people will die of drug-resistant bacterial infections every year by 2050. In the face of such a severe situation, on one hand, it is necessary to accelerate the development of antibiotics and meanwhile avoid the abuse of antibiotics; on the other hand, it is necessary to develop new antibacterial strategies.

Nanoparticle-mediated physical stimulation therapy is a promising bacterial therapy strategy that can partially replace antibiotics (Ji et al., 2022; Wang et al., 2022). In such a strategy, special nanoparticles are utilized as antennas to convert physical stimulation (e.g., light, magnetic, X-ray, and ultrasound) into heat energy or free radicals for bacterial inactivation (Jia and Zhao, 2021; Zhang et al., 2022b; Deng et al., 2022; Ji et al., 2022). For example, most of the noble metal nanoparticles, nano-carbon materials, magnetic nanomaterials, some nanopolymers, etc. can be heated under light, magnetic, ultrasonic, or other physical stimulations to generate high temperature for bacterial inactivation; photosensitizers and nanosemiconductor materials (such as titanium dioxide, bismuth vanadate, and quantum dots) can generate free radicals under light, X-ray, or even ultrasound irradiation for bacterial disinfection (Karami et al., 2021; Du et al., 2022; Fan et al., 2022). Among these strategies, the photothermal strategy has obvious advantages such as easy access to light sources, high bacterial inactivation efficiency, and low toxic side effects. In addition, photothermal treatment is not easy to induce bacterial resistance (Zhang et al., 2020; Mu et al., 2022). Therefore, in recent years, photothermal antibacterial therapy has attracted wide attention, and many progresses have been achieved in this field (Han et al., 2020). Achieving high antibacterial efficiency is no longer a major problem of photothermal methods. Improving the accuracy of treatment is currently a development trend in this field. Although modifying targeted molecules such as antibodies and antimicrobial peptides can improve the accuracy of nanophotothermal therapy to a certain extent, the modification also brings high cost problems. Using the special microenvironment of the lesion site to construct a responsive photothermal treatment strategy is an effective means to improve the accuracy of treatment. Such a strategy is expected to be a good solution to improve the accuracy of nanoparticle-mediated photothermal therapy.

**SCHEME 1:**
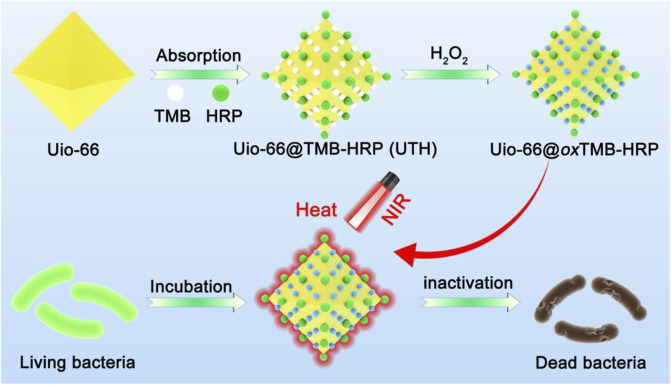
Schematic illustration of the preparation and antibacterial mechanism of the nanocomposite.

In this study, a metal–organic framework (MOF) nanocomposite responding to the oxidation state of the bacterial infection microenvironment was constructed for near-infrared (NIR) photothermal bacterial inactivation. In this strategy, MOF (UiO-66) was used as a nanocarrier to load tetramethylbenzidine (TMB) and horseradish peroxidase (HPR) (Scheme 1). The high oxidation state of the bacterial infection microenvironment can trigger the enzyme-catalyzed reaction of the nanocomposite, thereby generating oxidation products with the NIR photothermal effect for bacterial disinfection.

## Materials and methods

### General information

1,4-Dicarboxybenzene, 70% zirconium propoxide [Zr(OnPr)4] solution in 1-propanol, and tetramethylbenzidine (TMB) were purchased from Shanghai Aladdin Bio-Chem Technology Co., Ltd. Horseradish peroxidase (HPR) was purchased from Sigma-Aldrich. The transmission electron microscopy (TEM) image was captured with a 120-KV JEM-1400 microscope with a Gatan Rio16 digital camera. The sample for TEM was prepared by dropping the dilute UiO-66 solution onto carbon-coated copper grids. Powder X-ray diffraction (XRD) patterns were recorded with a Bruker D8 diffractometer (Bruker, Germany). UV-vis adsorption spectra were detected using a UV-1900 spectrometer (SHIMADZU, Japan). Fluorescence images were captured using a NIB900-FL fluorescent microscope with a Nexcan-T6CCD digital camera (Nexcope, China). A homemade 900-nm NIR light source was used for NIR light irradiation, and the power density was measured with a power density meter. A colony counter icount 11 (Xun Shu, China) was used to count colony-forming units.

### Synthesis of the metal–organic framework and the nanocomposite

MOF UiO-66 was synthesized according to the previous literature with some slight modifications (DeStefano et al., 2017). A measure of 3.5 ml of DMF, 2 ml of acetic acid (2.1 g, 35 mmol), and 30.5 μl of a 70% zirconium propoxide [Zr(OnPr)4] solution (in 1-propanol) (26 mg, 0.079 mmol) were mixed in a 10-ml scintillation vial. The solution was heated in an oil bath at 130°C for 2 h and then allowed to cool to room temperature. The color of the mixture changed from colorless to yellow during heating. To the solution, 37.5 mg of 1,4-dicarboxybenzene was added, and after sonication for 30 s, the solution was stirred at room temperature for 18 h. Then, the MOF was separated by centrifugation and washed several times with DMF and water and finally dispersed in water for further use. The nanocomposite (UiO-66@TMB-HRP, UTH) was synthesized by simple incubation of UiO-66 with TMB and HRP. Briefly, to a 5 ml solution of 5 mg/ml UiO-66, the TMB stock solution was added with a final concentration of 0.5 mM; after incubation for 3 h, 25 U HRP was added and stirred at 4°C for another 8 h. After then, the nanocomposite was separated by centrifugation, washed several times with water, and finally dispersed in water for further use.

### Photothermal measurement

The photothermal effect of UTH in different conditions under 900 nm light (0.5 w/cm^2^) irradiation was measured by using a thermal imaging camera. The heating and cooling temperature changes were recorded, and the photothermal conversion efficiency (*η*) was calculated according to the following equations:

$$\eta =\frac{hs\left(T_{\max}-T_{suur}\right)-Q_{0}}{I\left(1-10^{-A_{900}}\right)},$$ (1)

$$\tau_{s}=\frac{m_{d}c_{d}}{hs},$$ (2)

$${Q_{s0}=hs(T}_{\max.water}-T_{suur}),$$ (3)

where *τ*_s_ was observed by linearly fitting the plot of the cooling time versus −Lnθ. *m*_*d*_ is the mass of the UTH solution, and *C*_d_ is the heat capacity of water (4.2 J g^−1^ K^−1^). *T*_max_ is the equilibrium temperature; *T*_surr_ is the surrounding ambient temperature; *T*_max, water_ is the maximum temperature of the heated water.

### Antibacterial test

*Escherichia coli* (*E. coli*) and *Staphylococcus aureus* (*S. aureus*) were selected as Gram-positive and -negative model strains, respectively. Monocolonies of the bacteria on a solid agar plate were transferred to 2 ml LB medium and shaken under 150 rpm at 37°C for 12 h. In the photothermal antibacterial experiments, the bacterial solution (with an optical density at 600 nm of 0.5) was mixed with UTH and 1 mM H_2_O_2_ was added; after incubation for 5 min, the mixture was irradiated under 900-nm light for another 5 min. After then, the treated bacterial solution was diluted and transferred to the solid agar plate. After being placed in an incubator at 37°C for 12 h, the plates were photographed and colonies were counted.

### Therapeutic effect against mouse skin wound infection

Kunming mice were used for skin wound infection model fabrication, which has been approved by the Ethics Committee of Animal Experiments in Zhejiang Sci-Tech University, and all procedures followed the guidelines for animal experiments in Zhejiang Sci-Tech University. The hair of the mouse’s quilt was removed with depilation cream. A small piece of the back skin was cut off to construct a wound model. A measure of 10 μl of the *S. aureus* solution with the OD600 of 1 was dropped to the wound for the infection. Then, 10 μl of UTH was dropped to the infected wound once a day for the first 3 days, followed by irradiation with 900-nm light for 5 min. Photographs of the wound were taken every day to record the changes.

## Results and discussion

### Synthesis and characteristics

**FIGURE 1:**
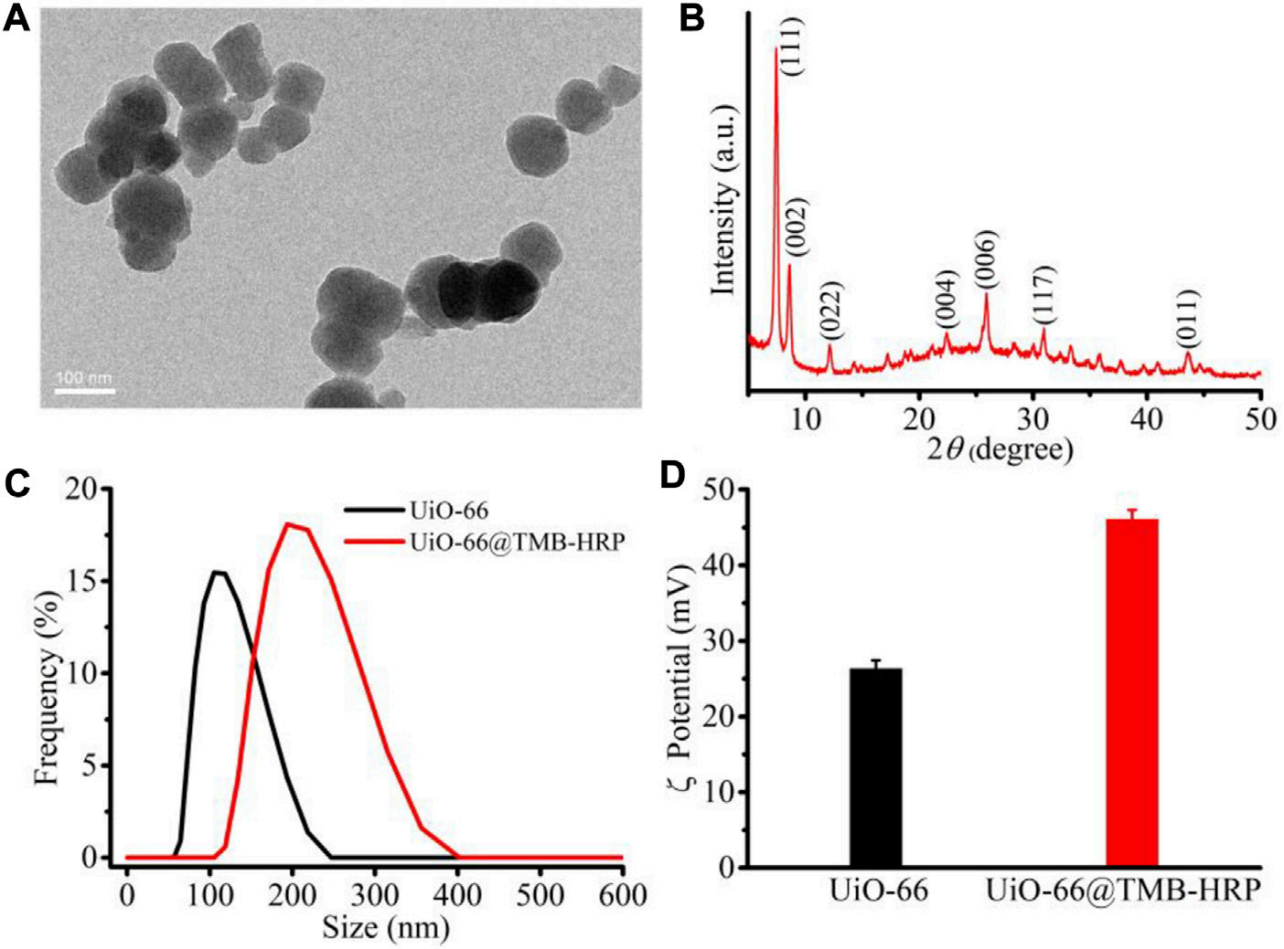
Characteristics of the nanocomposite preparation. TEM **(A)** image and XRD patterns **(B)** of the MOF (UiO-66), the size distribution **(C)**, and zeta potential **(D)** of the MOF and the obtained nanocomposite UiO-66@TMB-HRP (UTH).

Considering the porous characteristics and high stability, zr-mof (UiO-66) was chosen as the nanocarrier for TMB and HRP loading. The prepared UiO-66 is milky white and has good stability and dispersion in water. TEM characterization results show that UiO-66 has a size of about 100 nm and good dispersion (Figure 1A). The crystalline and phase information were investigated by powder X-ray diffraction (XRD), and the patterns are shown in Figure 1B. The appearance of sharp peaks in the XRD patterns indicates that UiO-66 has good crystallinity. The size and zeta potential of UiO-66 and UiO-66 loaded with TMB and HRP (UiO-66@TMB-HRP, UTH) were measured with a DLS machine. The main size was around 110 nm; after TMB and HRP loading, the obtained nanocomposite UTH showed a larger size of around 210 nm (Figure 1C). UiO-66 shows a positive zeta potential of 26.3 mV; after loading with TMB and HRP, the zeta potential increased to 46.1 mV (Figure 1D). The positive zeta potential of UiO-66 is ascribed to the positive charge of the Zr^4+^ cation. Both TMB and HRP are positive structures; therefore, after the loading, the zeta potential of the nanoparticles increased to 46.1 mV. The pore structure and the intermolecular interactions of aromatic molecules are mostly responsible for the loading of TMB. The coordination between the Zr^4+^ cation of UiO-66 and chelating groups (e.g., −COOH and −SH) of HRP would be the reasons for HRP loading. The changes in the size and zeta potential clearly indicated the successful preparation of the nanocomposite UTH. Moreover, the presence of the characteristic peaks of TMB and HRP in the absorption spectrum of the nanocomposite also indicated the successful loading of TMB and HRP by UiO-66 (Supplementary Figure S1).

### Response characteristics to H_2_O_2_

**FIGURE 2:**
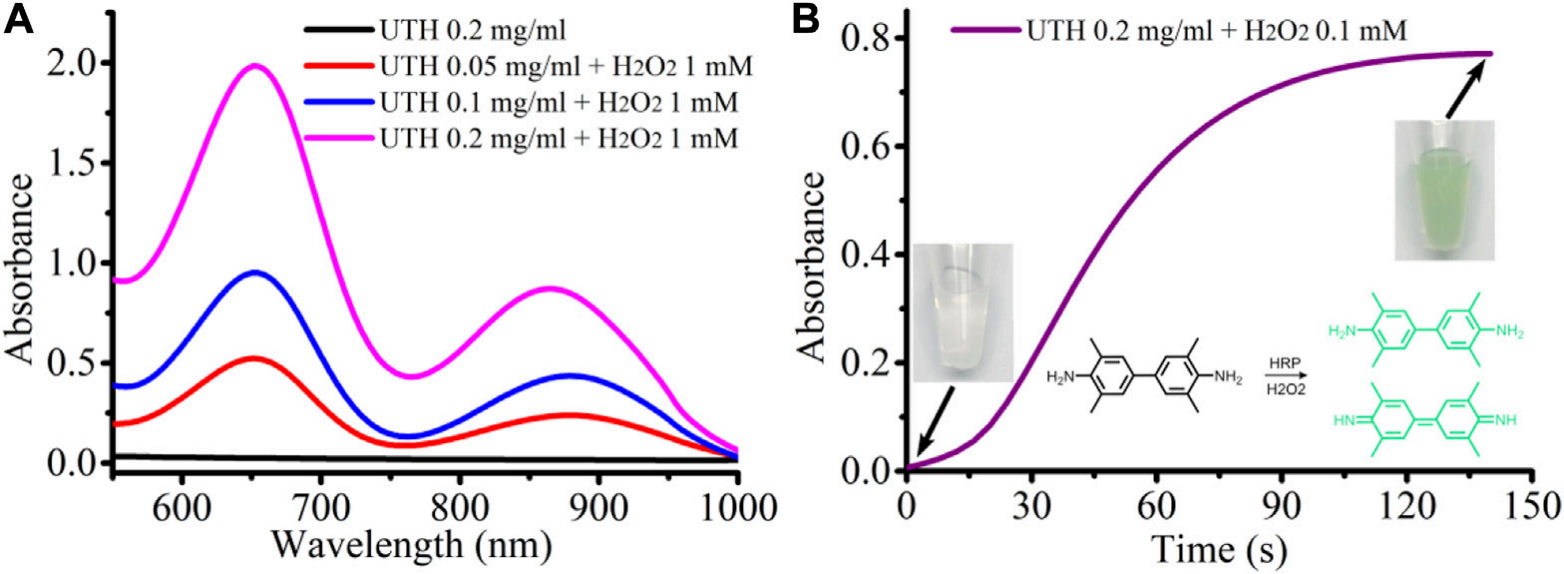
**(A)** Absorption spectra of the UTH before and after incubation with H_2_O_2_. **(B)** Change of the absorption value at 650 nm with time in the UTH–H_2_O_2_ system.

Then, we studied the response characteristics of the nanocomposite to H_2_O_2_ (Figure 2). The nanocomposite UTH contains both the enzyme (HRP) and the substrate (TMB); in the presence of H_2_O_2_, the HRP will catalyze H_2_O_2_ to generate an intermediate that can oxidize TMB to a colored state. As expected, the color of the nanocomposite solution changes from light milky to dark turquoise, and the absorption spectra clearly indicate the generation of the oxidized product of TMB (Figure 2A). After being treated with H_2_O_2_, two strong absorption peaks around 650 nm and near 900 nm appeared. The intensity of the absorption peak increases with the increase in the concentration of the nanocomposite (Figure 2A). Even when the concentration of H_2_O_2_ is as low as 0.1 mM, it can obviously cause discoloration of the nanocomposite in a short time, indicating that the nanocomposite has a high sensitivity to H_2_O_2_ (Figure 2B). It is worth mentioning that the photothermal effect of nanomaterials is often closely related to the intensity of their absorption peaks, which means that the oxidized nanocomposites will likely produce a strong photothermal effect under near-infrared light (900 nm) irradiation.

### Photothermal effect

**FIGURE 3:**
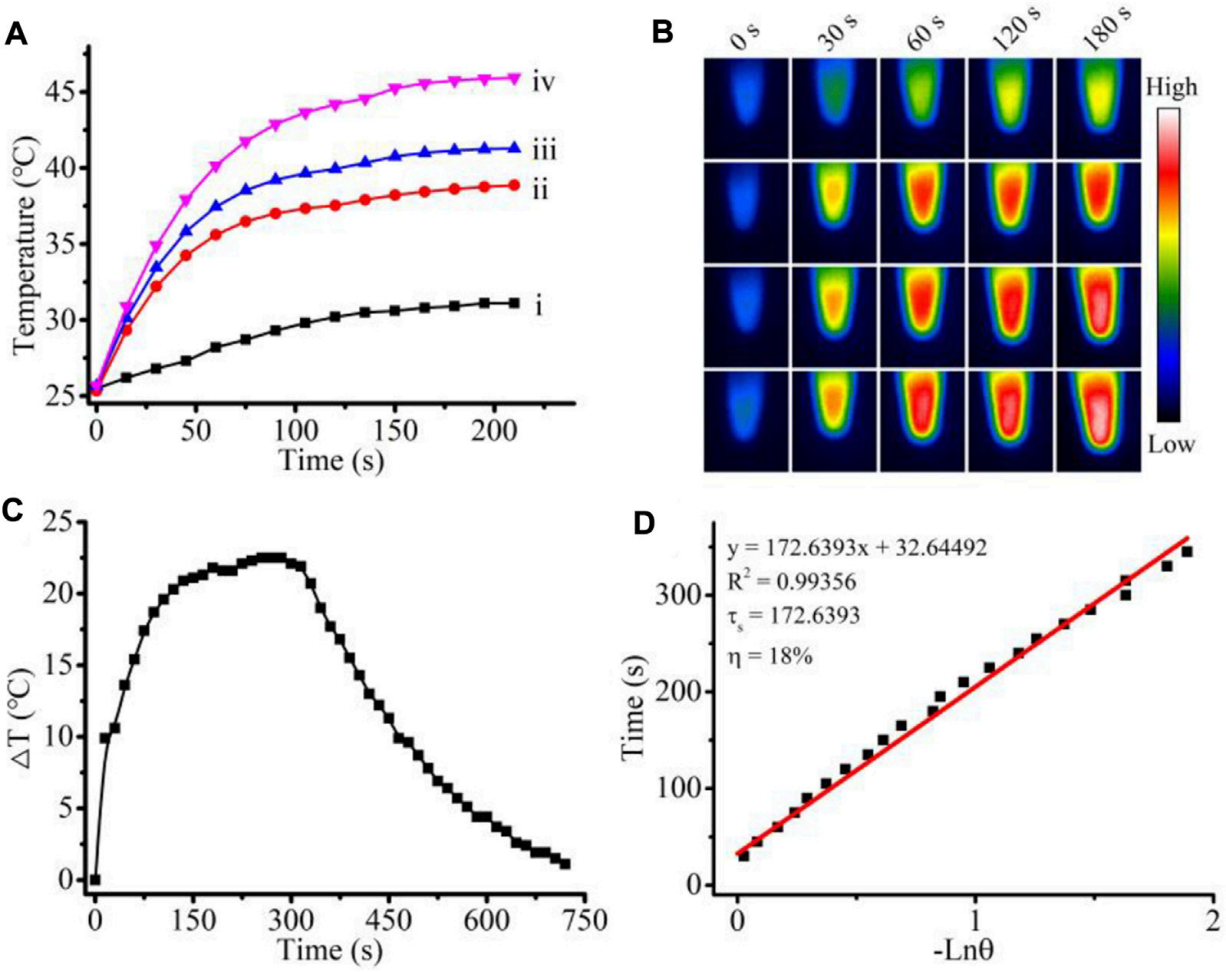
**(A)** Photo thermal effect of the UTH 0.2 mg/ml (i), UTH 0.05 mg/ml with 1 mM H_2_O_2_ (ii), UTH 0.1 mg/ml with 1 mM H_2_O_2_ (iii), and UTH 0.2 mg/ml with 1 mM H_2_O_2_ (iv) under 900-nm NIR light irradiation. **(B)** Corresponding thermal image in **(A)**. **(C)** “On-oﬀ” temperature change of UTH 0.2 mg/ml with 1 mM H_2_O_2_ under 900-nm light irradiation. **(D)** Liner cooling time data versus −Ln (θ) vs. negative natural logarithm of driving force temperature with τs = 172.63933 s.

As shown in Figures 3A,B, under 900-nm light irradiation, the solution of 0.2 mg/ml UTH showed a very slight temperature increase. However, in the presence of 1 mM H_2_O_2_, even a low concentration of UTH (0.05 mg/ml) could be sharply heated up by a 900-nm light irradiation. The heating rate and the maximum temperature increased with the increase in the concentration of nanocomposites. The temperature of the solution with 0.2 mg/ml UTH and 1 mM H_2_O_2_ can reach above 45°C within 3 min with 900 nm light irradiation, clearly indicating the excellent photothermal effect. It is worth mentioning that the concentration of H_2_O_2_ in the bacterial infection area is usually about 1 mM. In addition, during light irradiation, the local temperature of the nanoparticle surface is much higher than the solution temperature. These indicate that the nanoparticles provide the necessary basis for the sensitive response to H_2_O_2_ and efficient antibacterial activity in the infected area. Then, we calculated the photothermal efficiency by detecting the heating and cooling rates (Figures 3C,D), and the results showed that the photothermal efficiency of the nanocomposite UTH in the presence of H_2_O_2_ reached 18%. The photothermal conversion efficiency of most organic nanomaterials is between 20 and 50% (Li et al., 2020). Compared with these photothermal materials, the photothermal efficiency of 18% is slightly lower. Nevertheless, it is worth mentioning that indocyanine Green (ICG), as an organic molecule frequently used in photothermal therapy, has a photothermal efficiency of only 9% (Li et al., 2020). It indicates that 18% is enough for effective photothermal therapy.

### Antibacterial effect

**FIGURE 4:**
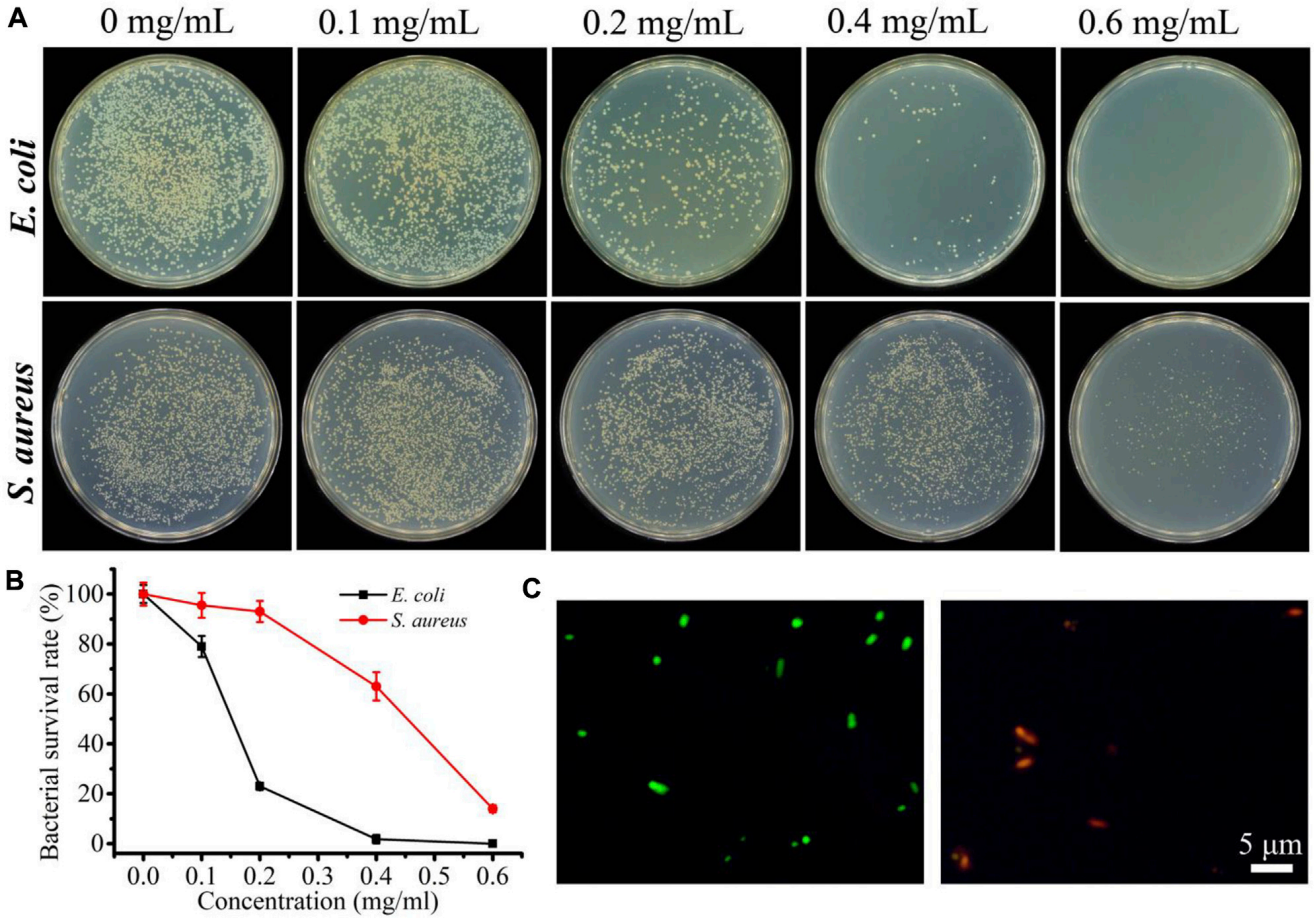
**(A)** Photograph of the colonies of *E. coli* and *S. aureus* treated with different concentrations of UTH in the presence of 1 mM H_2_O_2_ under 900-nm light irradiation. **(B)** Corresponding bacterial viabilities by counting **(A)**. **(C)** Fluorescence images (stained with SYTO 9 and PI) of *E. coli* before (left) and after (right) being treated with UTH in the presence of H_2_O_2_ under 900-nm light irradiation.

After verifying the sensitive response to H_2_O_2_ and the good photothermal effect of UTH, we then investigated the cascade antibacterial effect. We selected *E. coli* and *S. aureus* as Gram-negative and -positive bacterial models, respectively. The plate counting method was used to measure the antibacterial efficiency. The results showed that in the presence of only H_2_O_2_ (1 mM) or UTH (Supplementary Figure S2), NIR light irradiation could not cause significant antibacterial activity. However, in the presence of both H_2_O_2_ and UTH, NIR light irradiation can cause obvious antibacterial activity, and the antibacterial activity increases with the increase in the concentration of UTH. The *IC*_50_ values of UTH on *E. coli* and *S. aureus* under NIR light irradiation with 1 mM H_2_O_2_ were around 150 and 450 μg/ml, respectively (Figures 4A,B). The significant antibacterial effect was observed in both Gram-positive and -negative bacteria, indicating such a cascade nano-system has broad-spectrum antibacterial properties. Notably, the antibacterial effect of the photothermal system on the two stains is slightly different, and *E. coli* was more sensitive to the photothermal effect. The different antibacterial efficiencies may be related to the different structures of the two bacteria. *S. aureus* has a cell wall composed of peptidoglycan, which is relatively stable (Zhang et al., 2022a). However, the surface of *E. coli* is a cell membrane composed of phospholipid molecules, which is more fragile than the cell wall of *S. aureus*. Therefore, the photodynamic system has a stronger inactivation efficiency for *E. coli*. Subsequently, the antibacterial mechanism was primarily discussed by live/dead staining with a LIVE/DEAD bacterial viability kit. In such an assay, the green fluorescence (SYTO 9 dye) indicates the live bacteria and the red fluorescence (PI dye) indicates the cell wall-damaged dead bacteria. As shown in Figure 4C, most of the treated bacteria have strong red fluorescence, indicating that the cascade nano-system can cause cell wall damage to kill bacteria.

### Therapeutic effect against mouse wound infection

**FIGURE 5:**
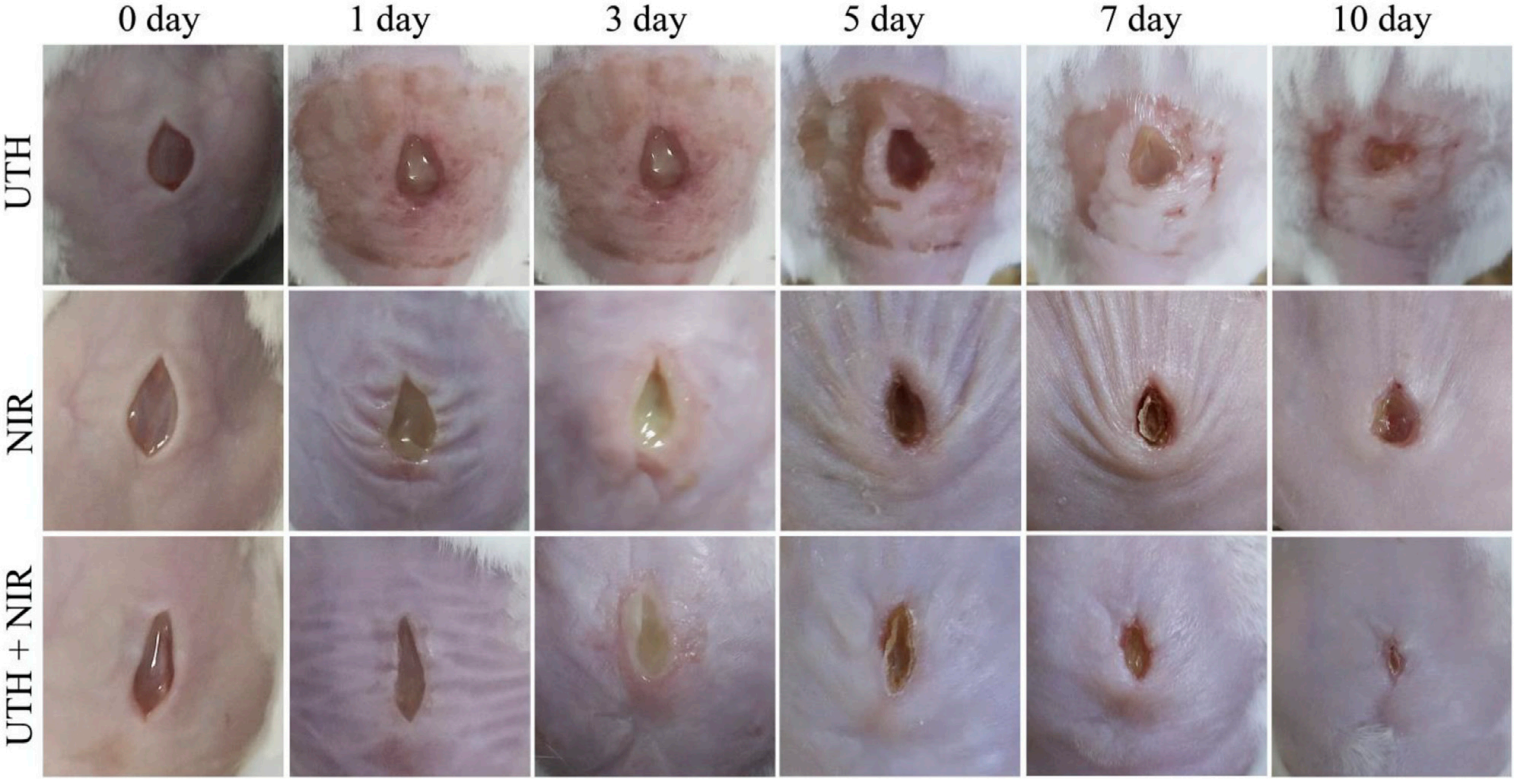
Therapeutic effect against mouse wound infection (caused by *S. aureus*) under different conditions.

Finally, in order to verify the therapeutic effect on real wound infection, we constructed a mouse skin infection model. As shown in Figure 5, after being treated by NIR light irradiation or UTH, the mouse skin wounds infected by *S. aureus* showed obvious symptoms such as suppuration, and the wounds healed slowly. Even 10 days later, the wounds still had obvious dents. However, after being treated with UTH and NIR light irradiation, the wound purulent symptoms were less and the wound healing was faster. After 10 days, the wound basically healed. These results show that the cascade nano-system has a good therapeutic effect on skin wound infection.

## Conclusion

In this study, the MOF nanocomposite with the characteristics of H_2_O_2_ and NIR light cascade response was successfully constructed, and the photothermal antibacterial activities were verified. The nanocomposites can respond sensitively and quickly to H_2_O_2_. Due to the catalytic oxidation, the color of the nanocomposite changes to dark turquoise in the presence of H_2_O_2_; meanwhile, a strong absorption peak in the near-infrared region around 900 nm appeared. The oxidized nanocomposite can convert near-infrared photons into thermal energy with an efficiency of 18%. This nano-system showed a strong inactivation effect on both Gram-negative and -positive bacteria. Under the conditions of 1 mM hydrogen peroxide and 0.5 W/cm^2^ NIR light intensity, the *IC*_50_ values of the MOF nanocomposite on *E. coli* and *S. aureus* were 150 and 450 μg/ml, respectively. This cascade response nanomedicine also showed a strong therapeutic effect on the mouse skin wound infection model. This study not only provides an effective photothermal antibacterial strategy but also offers a new idea for building precise nano-therapeutic systems.

## Data Availability

The original contributions presented in the study are included in the article/Supplementary material; further inquiries can be directed to the corresponding author.
