# Supplementary material for: A metal–organic framework nanocomposite with oxidation and near-infrared light cascade response for bacterial photothermal inactivation
Source: Front Chem. 2022 Oct 31;10:1044931. doi: 10.3389/fchem.2022.1044931 (PMC9667392; doi:10.3389/fchem.2022.1044931)
Supplement: Supplementary file 1 [file DataSheet1.PDF]

A MOF nanocomposite with oxidation and near-infrared light cascade response for bacterial photothermal inactivation

Momo Jr Christopher Dorma[1]†, Yuan Zhou,[2,3]†, Lanxin Li,[4,1]† Weisheng Zhu,[1] Luyao Wang,[4] Xingping Liu,[5] Wei Bing,[4] Zhijun Zhang[1]*

[1] Department of Chemistry, Key Laboratory of Surface & Interface Science of Polymer Materials of Zhejiang Province, Zhejiang Sci-Tech University, Hangzhou 310018, China.

[2] Department of Pharmacy, Taihe Hospital, Hubei University of Medicine, Shiyan, Hubei, 44200, China.

[3] College of Pharmacy, Hubei University of Traditional Chinese Medicine, Wuhan, 430065, China.

[4] School of Chemistry and Life Science, Changchun University of Technology, 2055 Yanan Street, Changchun 130012, China.

[5] School of Pharmaceutical Science, University of South China, Hengyang 421001, China.

†These authors contributed equally to this work.

*** Correspondence:**

Corresponding Author

zjzhang@zstu.edu.cn

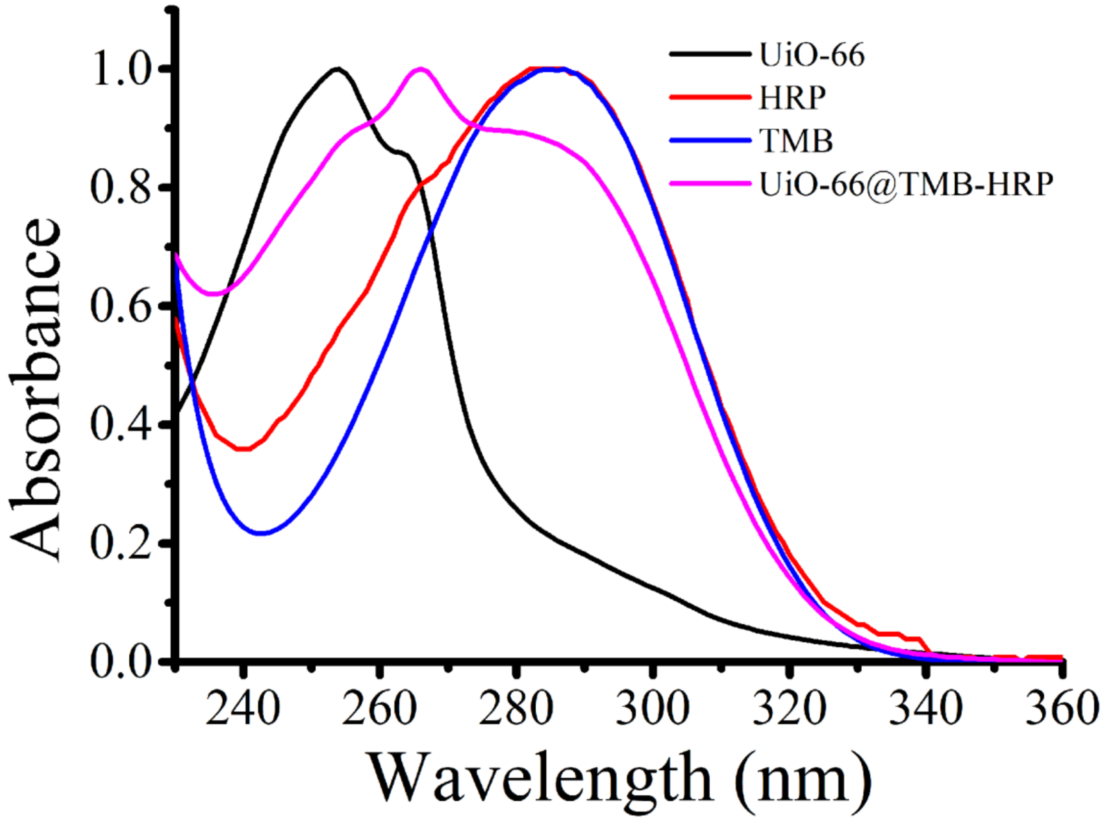

Fig. S1 Normalized UV-Vis spectra of UiO-66, HRP, TMB, and the obtained nanocomposite (UiO-66@TMB-HRP, UTH).

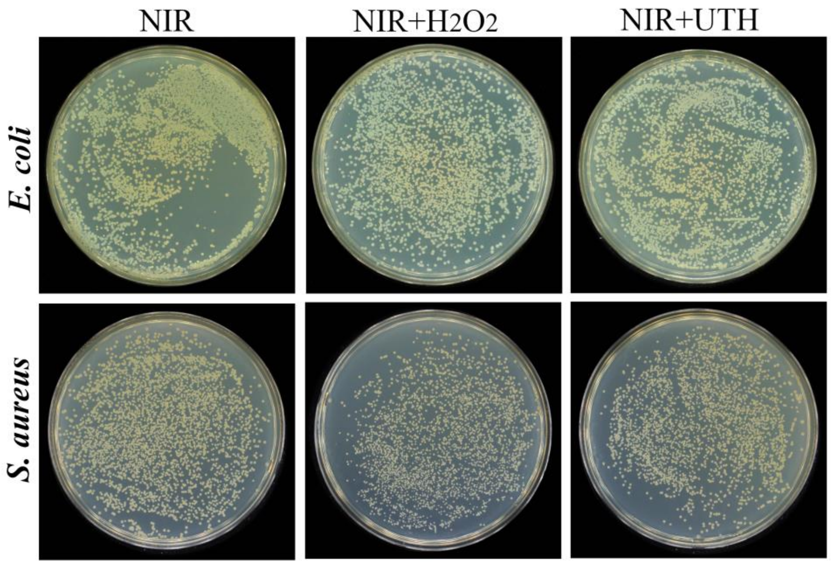

Fig. S2 The photograph of the colonies of *E. coli* and *S. aureus* treated under different conditions wiht 900 nm light irradiation. H_2O_2 1 mM, UTH 0.4 mg/ml.
